# KRAS: Biology, Inhibition, and Mechanisms of Inhibitor Resistance

**DOI:** 10.3390/curroncol31040150

**Published:** 2024-04-03

**Authors:** Leonard J. Ash, Ottavia Busia-Bourdain, Daniel Okpattah, Avrosina Kamel, Ariel Liberchuk, Andrew L. Wolfe

**Affiliations:** 1 Department of Biological Sciences, Hunter College, City University of New York, New York, NY 10065, USA; 2 Molecular, Cellular, and Developmental Biology Subprogram of the Biology Ph.D. Program, Graduate Center, City University of New York, New York, NY 10031, USA; 3 Biochemistry Ph.D. Program, Graduate Center, City University of New York, New York, NY 10031, USA; 4 Macaulay Honors College, Hunter College, City University of New York, New York, NY 10065, USA; 5 Department of Pharmacology, Weill Cornell Medicine, New York, NY 10021, USA

**Keywords:** KRAS, cancer, immunotherapy, targeted therapy, resistance, oncogene, inhibitors, mutation

## Abstract

KRAS is a small GTPase that is among the most commonly mutated oncogenes in cancer. Here, we discuss KRAS biology, therapeutic avenues to target it, and mechanisms of resistance that tumors employ in response to KRAS inhibition. Several strategies are under investigation for inhibiting oncogenic KRAS, including small molecule compounds targeting specific KRAS mutations, pan-KRAS inhibitors, PROTACs, siRNAs, PNAs, and mutant KRAS-specific immunostimulatory strategies. A central challenge to therapeutic effectiveness is the frequent development of resistance to these treatments. Direct resistance mechanisms can involve KRAS mutations that reduce drug efficacy or copy number alterations that increase the expression of mutant KRAS. Indirect resistance mechanisms arise from mutations that can rescue mutant KRAS-dependent cells either by reactivating the same signaling or via alternative pathways. Further, non-mutational forms of resistance can take the form of epigenetic marks, transcriptional reprogramming, or alterations within the tumor microenvironment. As the possible strategies to inhibit KRAS expand, understanding the nuances of resistance mechanisms is paramount to the development of both enhanced therapeutics and innovative drug combinations.

## 1. Introduction

### 1.1. KRAS Structure and Function

**Figure 1:**
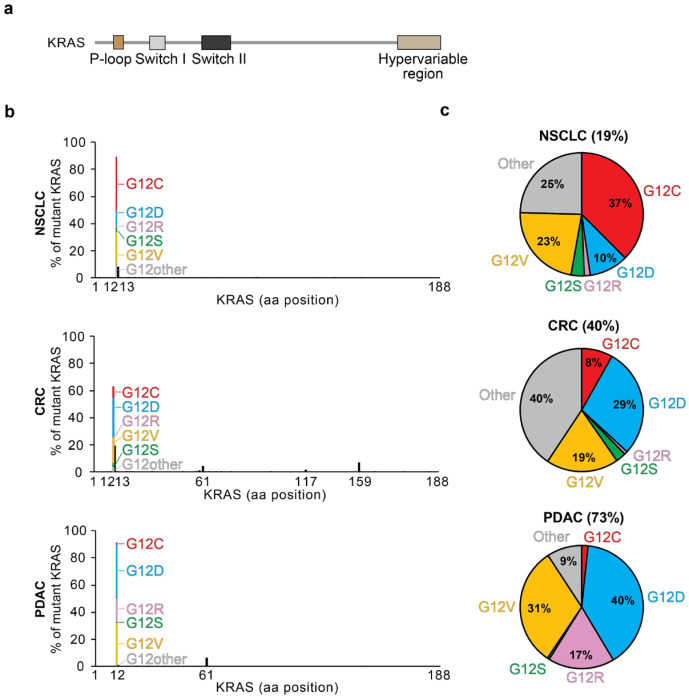
(**a**) Domain architecture of KRAS; (**b**) frequency of KRAS mutation by disease and location. Specific amino acid changes at G12 are color-coded by relative frequency. Mutation data were downloaded from the curated set of non-redundant samples in cBioportal [1,2]. NSCLC, non-small cell lung cancer; CRC, colorectal carcinoma; PDAC, pancreatic ductal adenocarcinoma; aa, amino acid. (**c**) Pie charts showing the frequency of KRAS mutations in non-small cell lung cancer, colorectal cancer, and pancreatic ductal adenocarcinoma. G12 mutations are color-coded as in (**b**). The other category represents KRAS mutations that are not G12C, G12D, G12R, G12V, or G12S.

KRAS is a small GTPase that ranks among the most commonly mutated oncogenic drivers in cancer. KRAS mutations are implicated in 25–30% of all human cancers, about 19% of non-small cell lung cancers (NSCLCs), about 40% of colorectal cancers (CRCs), and about 73% of pancreatic ductal adenocarcinoma (PDAC) cases (Figure 1) [1,2]. KRAS is a member of the RAS family of evolutionarily related genes, which also includes the commonly mutated HRAS and NRAS. The same genomic locus encodes for two alternatively spliced proteins, KRAS4A and KRAS4B, which are identical except for the final C-terminal hypervariable exon. RAS proteins are GTPase molecular switches that cycle between inactive, GDP-bound forms and active GTP-bound forms. The active forms bind to downstream effectors to stimulate their activity. KRAS is composed of two major structural regions: (1) the G-domain and (2) the C-terminal unstructured hypervariable region. The G-domain (codons 1–165) contains the switch I, switch II, and p-loop regions, which change conformation depending on the binding of GDP or GTP (Figure 1a).

### 1.2. KRAS Activity Is Regulated at Multiple Levels

**Figure 2:**
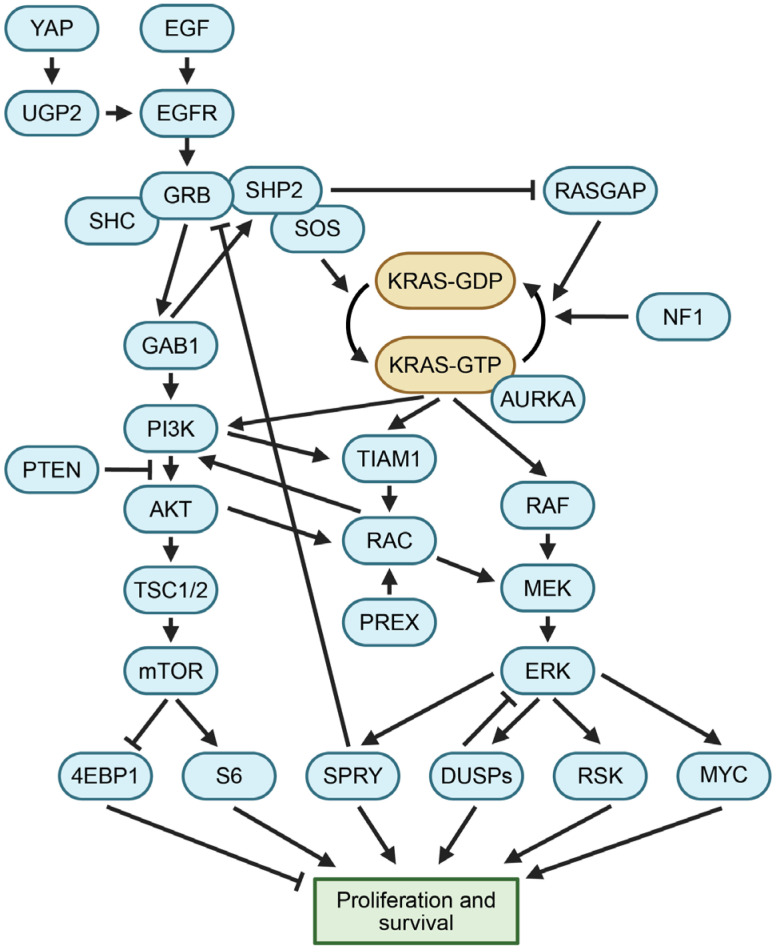
Diagram of signaling pathways related to KRAS. Growth signals stimulate KRAS to enter the active GTP-bound state. Downstream pathways, including PI3K and RAF proteins, translate active KRAS into signals for proliferation and survival. See the Glossary for acronym definitions.

After the KRAS protein is synthesized, post-translational lipid modifications are added to the C-terminal hypervariable region (HVR), targeting RAS proteins to the plasma membrane where they engage with other membrane-localized proteins [3,4]. This involves a prenylation step by FTase or GGTase, followed by subsequent modifications by RCE1 and ICMT. The palmitoylation of the HVR can act as a second signal promoting the membrane localization of RAS proteins [5,6]. Wildtype KRAS signaling is stimulated by upstream growth factors binding to receptor tyrosine kinases. For example, a stimulus cascade is initiated by EGF binding to its receptor EGFR, causing the activation of the cross-phosphorylation of dimerized EGFR (Figure 2). Activated EGFR leads to the phosphorylation of SHP2 and GRB2, resulting in the activation of SOS1 [7,8]. SOS1 functions as a guanine nucleotide exchange factor (GEF), which increases the efficiency of GTP loading on KRAS to favor its active state [9]. In contrast, GTPase-activating proteins (GAPs) like NF1 and p120-RasGAP catalyze the γ-phosphate hydrolysis of KRAS bound to GTP, increasing the proportion of inactive KRAS bound to GDP [10]. RGS enzymes are a family of GAPs that were recently characterized as favoring the catalyzation of the hydrolysis of mutant KRAS isoforms more than wildtype KRAS, indicating that KRAS mutations can create conformations with differential binding interactions, leading to alternative mechanisms of regulation in normal cells and cancer cells [11]. The phosphorylation of KRAS by PKC or PKG2 can change its protein interaction profile, promote the removal of KRAS from the membrane, and increase its rate of endocytic recycling [5,12,13].

### 1.3. KRAS Regulates Growth through Multiple Mechanisms

RAS proteins regulate essential signaling pathways that modulate growth and proliferation. Two of the most well-characterized downstream signaling cascades activated by RAS proteins are the mitogen-activated protein kinase (MAPK) and phosphoinositide 3-kinase (PI3K) pathways (Figure 2). In the MAPK cascade, two units of active KRAS-GTP bind to a dimer consisting of two units of either ARAF, BRAF, or CRAF, resulting in the activation of RAF kinase activity [14,15]. Subsequently, RAF phosphorylates MEK, which then phosphorylates ERK. Activated ERK, in turn, phosphorylates several cytoplasmic targets and also translocates to the nucleus, where it acts as a pro-growth transcriptional regulator that phosphorylates multiple nuclear transcription factors [14,16]. The genetic deletion of CRAF in established murine lung adenocarcinomas driven by mutant KRAS and p53 does not halt tumor growth [17]. This finding suggests that inhibitors of RAF alone are insufficient to treat KRAS-mutant cancers, given the diverse signaling pathways essential to KRAS function.

Active KRAS binds to and activates PI3K. This facilitates the conversion of PIP2 to PIP3, resulting in PDK1 phosphorylating AKT, which in turn activates mTORC1 via TSC1/2 inhibition and RHEB [18,19,20]. Moreover, KRAS can activate additional downstream signaling pathways. For example, RAL and TIAM1 have KRAS-binding domains. These effectors can be stimulated to activate RAC, a protein implicated in the control of cell motility and macropinocytotic processes [21,22,23]. Pathways downstream of KRAS ultimately promote proliferation and cell survival, which are key to the growth and maintenance of cancer cells when KRAS is inappropriately activated.

### 1.4. Impact of KRAS on Metabolic Processes

The rewiring of cellular metabolism is a well-recognized hallmark of cancer. Oncogenic KRAS has been identified as a key player in inducing significant metabolic changes in cells and can drive alterations in various metabolic pathways, including glycolysis, glutaminolysis, and micropinocytosis [24]. These altered metabolic pathways enable the cancer cells to grow and multiply more effectively. One of the main rewired metabolic pathways is glucose metabolism. Altered KRAS signaling reprograms cellular metabolism towards aerobic glycolysis. Oncogenic KRAS promotes the upregulation of the glucose transporter GLUT1, which enhances the uptake of glucose by cancer cells, in addition to other glycolytic enzymes such as HK1, HK2, and LDHA. The KRAS 4A splice isoform has been reported to directly interact with HK1 [25]. Mutant KRAS is involved in the rerouting of glycolytic intermediates into anabolic pathways such as the hexosamine biosynthesis, which regulates protein glycosylation, and the pentose phosphate pathway, which produces ribose for nucleotide synthesis [26]. Macropinocytosis is elevated in KRAS-mutated cancer cells to facilitate the bulk uptake of extracellular components. These include proteins that are then degraded into amino acids such as glutamine, which serves as a major carbon and nitrogen source for various metabolic pathways [22]. Notably, increased reliance on glutamine is a well-documented metabolic vulnerability in KRAS-mutant cancers [26]. Glutamine is transformed into alpha-ketoglutarate, which is essential for the anchorage-independent growth of cells driven by KRAS [27].

### 1.5. KRAS Mutations

Patterns inherent in KRAS mutations provide insight into how mutations arise, their specific function in different tissues, and their effector-binding affinities. KRAS mutations most commonly occur at the 12th codon near the nucleotide-binding site, with less prevalent mutations occurring at codons 13, 61, and 146 within other catalytic domains (Figure 1b,c) [23]. Mutations in KRAS can impact the intrinsic or GEF-mediated GDP/GTP exchange rate (Figure 2) [4,28]. They can also impact the intrinsic or GAP-mediated hydrolysis rate [4]. KRAS G12R, G13D, Q61H, Q61L, and A146T mutations have increased intrinsic exchange relative to wildtype, but G12C and G12V do not display large changes [4,29,30,31,32]. Most KRAS mutations are deficient for intrinsic hydrolysis; however, KRAS G12C maintains much of its intrinsic hydrolysis activity. These intrinsic properties can lead to biological differences between point mutations activating KRAS.

Specific KRAS mutations show characteristic patterns of tissue preference (Figure 1b,c). G12D and G12V mutations are the most common in PDAC, followed by G12R. KRAS A146 mutations are primarily found in colorectal cancer and are rarely reported in other types. G12C and G12V mutations are predominant in lung adenocarcinomas with smoking-associated mutational signatures [30,32]. One exogenous cause of the increased prevalence of G12C mutations in lung cancers is benzo[a]pyrene-diol epoxide (BPDE), a carcinogen found in cigarettes [33,34]. BPDE covalently binds to guanine to promote a transversion to thymine, which predisposes to either G12C or G12V mutations. Notably, lung adenocarcinomas in smokers exhibit a higher frequency of G to T transversions compared to those in nonsmokers [35].

Notably, KRAS G12R exhibits a reduced affinity for PI3K binding in PDAC relative to other KRAS G12D and G12V mutants, leading to impaired PI3K signaling [36]. Compared to other KRAS-driven diseases like lung adenocarcinoma and colorectal cancer, pancreatic cancer has a higher prevalence of G12R, consistent with MAPK being the dominant signaling pathway driving pancreatic cancer [36,37]. Furthermore, the distribution of somatic mutations is a contributing factor to disparities in cancer health outcomes, especially in the only currently clinically targetable KRAS G12C allele. Lung adenocarcinomas in Black or Asian patients tend to exhibit KRAS mutations other than G12C more frequently than White patients, who are more likely to have tumors driven by the clinically targetable KRAS G12C [38]. Thus, certain populations are underserved by current clinically approved G12C-selective therapeutic options.

Next, we discuss therapeutic strategies to inhibit KRAS and explore the mechanisms by which cancers develop resistance, whether through on-target, off-target, or non-mutational alterations.

## 2. Targeted KRAS Inhibitors

### 2.1. KRAS as a Therapeutic Target

As a frequently mutated oncogenic driver protein, KRAS represents a compelling therapeutic target for cancer treatment. The induced genetic deletion of mutant KRAS in the murine NSCLC tumor model Kras^+/FSFG12Vlox^;Trp53^F/F^;Rosa26-CreERT2^KI/KI^;Tg.hUBC-CreERT2^+/T^ caused rapid tumor regressions [39]. This mouse model employed two independent Cre loci to prevent the common resistance mechanism of Cre inactivation. Early indirect attempts to treat KRAS-mutant tumors included therapeutically exploiting the apparent requirement of the farnesylation of RAS proteins for localization to the plasma membrane using farnesyltransferase inhibitors (FTIs). The limited success of FTIs is largely attributed to the alternate prenylation of KRAS and NRAS by geranylgeranyltransferase [40]. Alternative compounds targeting enzymes that modify RAS proteins post-prenylation, ICMT and RCE1, have been tested and show anti-cancer activity in cell and animal models [41,42]. However, the lack of specificity or selectivity is a concern and mislocalized RAS may retain partial activity [43]. Several clinically approved inhibitors target molecules downstream of KRAS, including RAF, MEK, ERK, PI3K, AKT, and mTOR [44]. While these inhibitors are each approved against various malignancies, mutant KRAS is not typically a specific biomarker [45]. Clinical trials involving inhibitors against RAF and MEK have not received approval for treating KRAS-mutant cancers [46].

### 2.2. KRAS G12C-Selective Covalent Inhibitors

**Figure 3:**
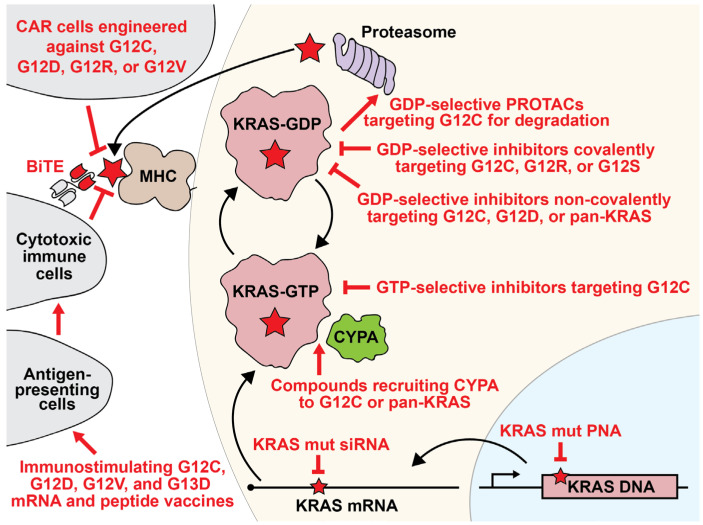
Schematic of categories of KRAS-focused strategies for inhibiting KRAS-mutant cancers. KRAS can be inhibited at the level of DNA, mRNA, protein, or as an antigen targeting the cell for immune destruction. Inhibitor strategies are shown in red. The red star represents an activating point mutation in KRAS. On the MHC, the red star represents an antigen containing a KRAS-mutant fragment. See the Glossary for acronym definitions.

In 2013, Kevan Shokat’s group introduced a groundbreaking class of compounds specifically designed to target KRAS G12C. These inhibitors covalently bind the reactive sulfhydryl residues on the mutated cysteine located at G12, rendering them specific to the G12C mutation [47]. Early iterations of this compound were selective for the GDP-bound state, effectively locking KRAS G12C in its inactive form (Figure 3) [47]. Notably, G12C retains GAP-independent hydrolysis activity, allowing GTP-bound KRAS G12C to cycle back to the more readily targetable GDP state [48]. Sotorasib and adagrasib, two prominent members of this class, were effective at halting the growth of G12C-mutant cancer cells in 2-dimensional, 3-dimensional, and xenograft models, clearing the path for clinical trials [48,49,50,51]. In 2021, the FDA approved sotorasib for the treatment of adult patients with KRAS G12C-mutated NSCLC, while adagrasib received similar approval the following year [48,50,51,52,53].

In addition to its direct inhibitory effect on KRAS-mutant cancer cells, sotorasib was found to promote antitumor immunity in a syngeneic CT-26 KRAS G12C colon cancer model by creating a pro-inflammatory tumor microenvironment [48]. Sotorasib administration led to the tumor infiltration of CD8 T-cells, macrophages, and dendritic cells, which were further amplified by the combination with anti-PD-1 therapy. Sotorasib treatment not only increased the intratumoral concentration of chemokines that promote immune infiltration but also induced the expression of neoantigens on MHC class I, leading to improved antigen recognition and potentially a more robust, long-lasting antitumor T-cell response. Notably, when mice treated with both sotorasib and anti-PD-1 to clear KRAS G12C tumors were rechallenged with KRAS G12D tumor cells, an immune memory response halted the growth of the tumor, highlighting the adaptive immune reactions to shared antigens [48]. This discovery gains significance considering the ability of KRAS G12D to boost GM-CSF production in PDAC cancer cells, thereby attracting immunosuppressive myeloid cells that can dampen the activity of CD8 T-cells [54]. These findings indicated the potential effectiveness of combining KRAS inhibitors with anti-PD-1/PD-L1 treatment, prompting further clinical trials.

While G12C-selective inhibitors represent the most promising clinical strategy to date, they come with significant limitations. More prevalent isoforms such as G12D and G12V do not have any approved targeted treatments. The high affinity of the cysteine-reactive warhead on KRAS G12C inhibitors has opened avenues for chemical refinements to optimize other interactions with nearby amino acids of KRAS. That optimization process led to compound structures with enough affinity for KRAS itself that the covalent warhead could be swapped for alternatives capable of binding G12D, G12R, G12S, or any KRAS-mutant allele.

### 2.3. KRAS G12D Inhibitors

Given the high prevalence of G12D mutations in cancer, there have been multiple attempts to create G12D-targeted inhibitors. Starting with the scaffold of Mirati’s G12C inhibitors, Mao et al. developed a series of G12D-selective inhibitor compounds [55]. The methyl-substituted piperazine inhibitor, the TH-Z8 series, works by forming a salt bridge interaction between the KRAS Asp12 residue and the compound’s alkyl amine moiety, inducing the formation of an allosteric drug-binding pocket. Notably, this molecule did not discriminate between GDP- and GTP-bound KRAS. The co-crystal structure of KRAS-TH-Z816 identified the compound’s binding site in an allosteric pocket induced in the KRAS G12D switch II region. This region overlaps with the pockets formed by the G12C inhibitor MRTX849, indicating that despite different warheads, they bind in a similar location. A variant compound significantly reduced tumor volume in KRAS G12D mouse xenograft models. However, this compound also displayed activity in off-target cell lines, suggesting other small GTPases such as Rho, Ran, Arf, and/or Rab may have also been targeted [55].

An alternative KRAS G12D inhibitor, MRTX1133, demonstrated KRAS G12D selectivity [56]. Like the TH-Z8 series, MRTX1133 binds both active and inactive KRAS G12D with high affinity. MRX1133 disrupted both PI3K and MAPK signaling in vitro and reduced tumor size in cell line-derived murine xenografts. This compound was shown to be most effective in inhibiting tumor activity in patient- and cell line-derived pancreatic G12D xenografts over xenografts from colorectal cancers and other tissues [57]. Clinical trials for MRTX-1133 against KRAS G12D solid tumors are ongoing [58].

### 2.4. KRAS G12S and KRAS G12R Tool Compounds

In 2022, the Shokat lab introduced a novel KRAS G12S-selective covalent inhibitor [59]. Compounds containing β-lactone electrophiles, designed to mimic the chemistry of natural proteasome inhibitors, covalently acylate the mutant serine of G12S in the switch II pocket. These compounds showed GDP-bound KRAS selectivity. These molecules were effective at halting the growth of cells expressing KRAS G12S at concentrations greater than 10 μM. Furthermore, the Shokat lab identified electrophiles with the potential for developing G12R-selective inhibitors [60]. Considering the success of the cysteine-targeting compound warhead approach originally devised for KRAS G12C and its subsequent application in targeting other proteins with mutant cysteines like Gαs, it is likely that novel compound chemistries capable of semi-selectively targeting serines or arginines may prove to have applications toward targeting both KRAS and other critical mutant proteins with point mutations [61].

### 2.5. Pan-KRAS Inhibitors

**Figure 4:**
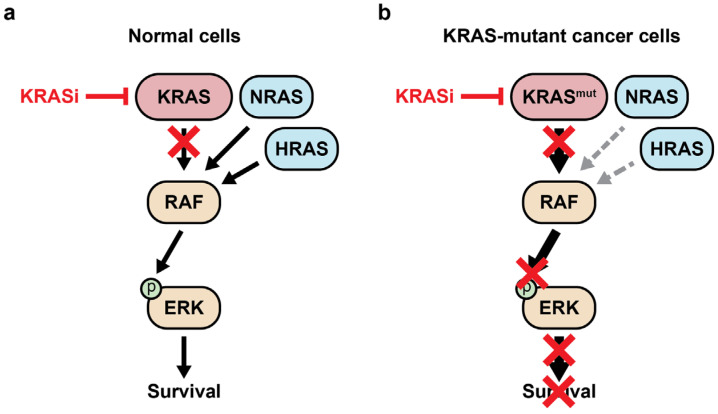
Model of RAS survival signals in wildtype and mutant KRAS cells. (**a**) In wildtype cells, KRAS, NRAS, and HRAS have overlapping functions regulating RAF activation. A pan-KRAS inhibitor may be tolerated because NRAS and HRAS can maintain normal survival signals. (**b**) KRAS-mutant cancer cells are rewired to be dependent on their continuous expression. The sudden inhibition of mutant KRAS rapidly decreases proliferation and survival signals, represented by red Xs. See the Glossary for acronym definitions.

The G12C inhibitor BI-0474 scaffold was used to develop the pan-KRAS inhibitor BI-2865, which non-covalently binds to KRAS-GDP with high affinity while sparing wildtypes HRAS and NRAS [62]. This inhibitor functions by blocking the GDP/GTP nucleotide exchange of wildtype KRAS and other KRAS mutants, including G12A, G12C, G12D, G12F, G12V, G12S, G13C, G13D, V14I, L19F, Q22K, D33E, Q61H, K117N, A146V, and A146T. BI-2865 inhibits oncogenic signaling through the RAF cascade, regardless of the specific KRAS driver mutation [62]. Murine xenograft experiments demonstrated efficacy against grafted cell lines and a PDX driven by KRAS G12C, G12D, G12V, and A146V without causing significant body weight loss. BI-2865 effectively blocked the interaction between KRAS and RAF in most cell lines, though it displayed weaker activity against cell lines harboring the KRAS G12R mutation. Interestingly, in KRAS wildtype cells, the compound was able to prevent the KRAS–RAF interaction; however, the inhibition of p-ERK and growth were markedly less pronounced compared to cells with mutant KRAS. The knockdown of HRAS and NRAS sensitized KRAS-wildtype cell lines to BI-2865 [63]. This observation supports the model that pan-KRAS inhibition might be well tolerated in normal cells due to functional redundancy provided by HRAS and NRAS, whereas mutant KRAS reprograms cell signaling to create a dependency on the continuous hyperactivation of the pathway (Figure 4).

### 2.6. Cyclophilin A Recruitment

An alternative strategy targeting mutant KRAS G12C involves molecular glue compounds that create a complex between mutant KRAS G12C and a non-canonical binding partner cyclophilin A (CYPA) [64]. The CYPA recruiter RMC-6291 forms an inhibitory tri-complex with KRAS G12C and CYPA, resulting in a steric blockade of effector activation, including RAF. In contrast to sotorasib and adagrasib, which lock KRAS in the inactive conformation, RMC-6291 binds active KRAS-GTP. In xenograft models, RMC-6291 outperformed adagrasib in reducing tumor volume, which paved the way for clinical trials in patients with KRAS G12C-driven tumors. Additionally, a pan-KRAS CYPA-recruiting compound, RMC-6236, is currently undergoing clinical trial [64].

### 2.7. siRNAs

Small interfering RNAs (siRNAs) can inhibit target messenger RNAs. Exogenous siRNAs are delivered into cells as synthetic complementary RNA strands encased in lipid nanoparticles [65]. Each siRNA is then processed by the endogenous RNA-induced silencing complex prior to binding to the target RNA, in this case, KRAS, thereby inhibiting translation and promoting mRNA degradation [66,67,68]. Early anti-KRAS siRNAs such as AZD-4785 targeted all KRAS isoforms; however, the trial for AZD-4785 was discontinued [69]. Off-target effects are concerning for siRNA-based therapies, as siRNAs can bind mRNAs despite one or two sequence mismatches, which may lead to generalized cell toxicity even in healthy tissue [66]. Alternative KRAS siRNAs have been designed to exploit mismatch binding to increase mutant KRAS specificity, by creating siRNA sequences containing three mismatches against wildtype but only two mismatches against three KRAS mutants: G12C, G12D, and G13D. This siRNA had a higher affinity for the targeted oncogenic mutations than wildtype [66]. Other siRNAs have been designed to specifically target KRAS G12D, such as siG12D-LODER, which is in clinical trials against KRAS G12D-driven pancreatic cancer [70].

### 2.8. Peptide Nucleic Acids

The transcriptional inhibition of mutant KRAS is an orthogonal approach to the methods described above. Engineered peptide nucleic acids (PNAs) have protein-like backbone structures bound to DNA-like bases. The avidity of PNAs for a single strand of matching DNA is higher than DNA itself. This allows PNAs to outcompete the antisense strand to selectively hybridize with the mutant region of the genome, thus obstructing transcriptional machinery and causing reduced expression [71]. PNAs have the advantage of being highly sequence-selective, enabling the targeting of mutant alleles while sparing the wildtype [72]. PNAs targeted against non-G12C alleles can be employed as a model for studying resistance to future non-G12C KRAS therapeutics.

### 2.9. Destabilizing and Degrading Agents

Proteolysis-targeting chimeras (PROTACs) are bifunctional molecules designed to simultaneously engage a protein of interest and the cell’s ubiquitination machinery, facilitating the ubiquitination of the target protein [73,74]. Once ubiquitinated, the target protein is then degraded by the proteasome. This approach combines the selective targeting capacity of drug molecules and leverages the cell’s intrinsic proteasome degradation systems, thereby circumventing the issue of catalytic inefficacy [75]. LC-2 is one such PROTAC that fuses the G12C selective inhibitor MRTX849 with the E3 ubiquitin ligase VHL [76]. Treatment with LC-2 resulted in the proteasome-dependent depletion of mutant KRAS and the reduction in downstream signaling [76]. PROTAC-based inhibitors show potential as an adaptable therapeutic strategy.

Synthetic amyloidogenic peptides, termed Pept-ins, represent a novel approach developed to induce the misfolding and aggregation of mutant KRAS, moving beyond the reliance on conventional binding pockets [77]. Oncogenic mutations at positions 12 and 13, especially the G12V mutation, enhance KRAS’s natural tendency towards aggregation. Pept-ins engineered to interact with these aggregation-prone regions have been shown to induce the misfolding and aggregation of KRAS, undermining its oncogenic function. Pept-ins have been assessed both in vivo and in vitro, demonstrating that they can interrupt critical signaling pathways, prompt apoptosis, and suppress tumor proliferation.

## 3. KRAS-Dependent Immunotherapies

### 3.1. Mutant KRAS Vaccines

Vaccination can train the immune system to attack cells expressing mutant KRAS. These vaccines are designed to induce antigen-presenting cells to display mutant KRAS, stimulating attacks by cytotoxic T-cells against cancer cells expressing mutant KRAS neoantigen [78,79,80]. A phase 2 clinical trial is investigating the delivery of KRAS-mutant neoantigens along with immune-activating checkpoint inhibitors in KRAS-mutant solid tumors as a method of training the immune system to target KRAS-mutant cells [81].

Building on the momentum of KRAS-specific cancer vaccines, a recent study introduced an innovative intranasal vaccine targeting NSCLC based on a mix of G12D mutant and wildtype KRAS peptides and enhanced by a nanoemulsion adjuvant [82]. This strategy not only aims to direct the immune system’s attention to KRAS mutations but also leverages the specificity of mucosal immunity to effectively combat tumors in the lung. This approach showed promise in a transgenic mouse model, where repeated vaccinations before and after the induction of KRAS G12D-driven lung tumors elicited a strong immune response. Immunized mice had increased CD4+ T-cell activity, a reduction in immunosuppressive regulatory T-cells, and higher levels of antigen-specific IFN-γ and IL-17 cytokines, primarily produced by CD8+ T-cells. The vaccine reduced tumor incidence and burden, representing a promising step forward in preventative immunization against KRAS-driven cancers.

mRNA-based vaccines have dominated public awareness throughout the course of the COVID-19 pandemic, and the 2023 Nobel Prize in Physiology or Medicine was awarded for the underlying discoveries that enabled their development. The landmark approval of COVID-19-targeted mRNA vaccines mRNA-1273 and BNT162b2 paved the way for cancer-focused mRNA vaccine strategies. Prior to the pandemic, Moderna used technology similar to the SARS-CoV-2 vaccine to develop mRNA-5671/V941, a tetravalent mutant KRAS mRNA vaccine containing sequences G12C, G12D, G12V, and G13D [83]. mRNA-5671/V941 is currently in Phase I clinical trials against KRAS-mutant NSCLC, PDAC, and CRC [84].

### 3.2. Bispecific T-Cell Engagers

Bispecific T-cell engagers (BiTEs) are a class of antibody-like biotherapeutics designed with dual specificity (Figure 3). By linking two distinct single-chain variable fragments (scFvs), BiTEs target both a tumor-associated antigen and a TCR component necessary for T-cell activation, typically CD3 [85]. This configuration allows BiTEs to attract cytotoxic T-lymphocytes to tumor cells, activating the T-cells to eliminate the cancerous targets. Notably, these BiTEs can bypass the traditional MHC class I molecule antigen presentation. In recent studies, teams from Zhang et al. and Hattori et al. developed specific BiTEs to tackle G12Ci-resistant cells [86,87]. The Zhang team developed a BiTE targeting ARS1620-modified peptides. This particular construct elicited a cytotoxic T-cell response against KRAS G12C cells, including those cells resistant to direct KRAS G12C inhibition. The Hattori group utilized the “HapImmune” platform to create BiTEs targeting sotorasib-conjugated peptides. Remarkably, the HapImmune-derived BiTE demonstrated the ability to effectively target and eliminate lung cancer cells that were resistant to sotorasib when exposed to the drug. These findings emphasize the potential of engineered immune antibody strategies for selectively targeting G12Ci-resistant cells, hinting at their therapeutic potential in precision tumor therapy.

### 3.3. Adoptive Immunotherapies

Tumors with mutant KRAS can express neoantigen fragments containing the oncogenic driver mutation on the major histocompatibility complex [88,89]. KRAS neoantigens can act as a tumor-selective biomarker that can be targeted using engineered T-cells [90]. Adoptive T-cell therapy has emerged as a promising approach for the treatment of KRAS-mutant cancers. In this therapy, CD8+ T-cells engineered to specifically target antigenic mutant KRAS fragments displayed by cancer cells are infused into patients. This approach has shown efficacy in colon cancer and NSCLC patients with mutant KRAS G12D and HLA-C*08:02 expression [88]. In a separate study, Wang et al. engineered an HLA-A11:01-restricted murine T-cell receptor (mTCR) that specifically recognizes G12D- and G12V-mutated KRAS. These studies led to Phase I/II clinical trials looking to establish the safety and effectiveness of PBL transduced with anti-KRAS G12D or anti-KRAS G12V mTCR in HLA-A*11:01 positive patients with advanced solid tumors expressing G12D or G12V mutated KRAS [91,92].

An alternative approach employs dendritic cells (DC) engineered against mutant KRAS epitopes. DCs engineered against mutant KRAS were efficacious when injected into mouse models of NSCLC, and a clinical trial applying this principle to humans has entered Phase I targeting KRAS G12C, G12D, G12R, and G12V [93].

This variety of strategies to inhibit mutant KRAS has the potential to positively impact a wide array of patients, many of whom do not currently have access to effective targeted treatment. As these new therapies become available, understanding their mechanisms of resistance intrinsic to each strategy will be essential for successful patient outcomes. Next, we discuss the methods and strategies cancers employ to resist KRAS inhibition.

## 4. Mutational Resistance

### 4.1. Resistance to KRAS Inhibitors

The majority of cancer treatments do not result in permanent cures. Clinical evaluations of KRAS inhibitors, sotorasib and adagrasib, were conducted as basket trials and included solid tumors with KRAS G12C regardless of the tissue of origin. The data from both CodeBreak 100 for sotorasib and KRYSTAL-1 for adagrasib led to the approval of these inhibitors to treat G12C-mutant NSCLC [51,53,94]. However, neither of these compounds was approved as single agents against colorectal cancer or other types of cancer with KRAS G12C due to subpar objective response rates. Even in NSCLC, where the compounds were approved, median response rates were in the 6–7 month range before relapse occurred, indicating that the vast majority of tumors develop resistance to single-agent KRAS G12C inhibitors [51,95]. Relative to NSCLC cell lines, CRC lines have higher basal RTK activation and higher rebound of p-ERK, suggesting one possible explanation for the higher KRASi sensitivity in lung tumors [96].

Treatment with a targeted KRAS inhibitor frequently selects for mutations that provide treated cells with a selective advantage, leading to drug resistance. Cells containing mutations conferring KRASi resistance are frequently clonally or subclonally selected and can come to dominate much of the tumor post-relapse [62,68,97]. Additionally, tumor genomes can become highly unstable as a result of impaired DNA damage repair or DNA proofreading functions, giving rise to spontaneous mutations throughout a cancer’s lifespan [98]. Furthermore, as tumors metastasize, their genomes significantly change under new selective pressures, altering their treatment sensitivities accordingly [99]. Studies of pre-treatment and post-treatment samples identify multiple mechanisms of tumor evolution both via low-frequency pre-existing mutations that expand after treatment and/or previously undetectable mutations that are induced by therapy de novo [99,100].

The treatment of cancer cells with KRAS inhibitors selects for two major classes of mutations: (1) alterations in KRAS itself which change its ability to bind the drug, its ability to bind to KRAS regulators, or its ability to bind to KRAS effectors, or (2) secondary mutations elsewhere in the tumor genome.

### 4.2. Drug Resistant Mutations to KRASi

Understanding the nature and frequency of on-target mutations in KRAS is likely to influence the structure of the next generation of KRAS inhibitors. Post-treatment analysis of NSCLC and CRC samples from patients treated with either sotorasib or adagrasib identified de novo oncogenic mutations secondary to treatment, including multiple amplifications and point mutations in KRAS G12C itself [101]. Point mutations in the KRAS gene included G12D/F/R/V/W, which prevent covalent interactions with mutant cysteine, as well as V8L, G13D, V14I, Q61H, H95D/Q/R, Y96C, and Y96D [62,97,102,103]. Modeling demonstrated that many of these mutations interfere with the binding site of the inhibitor [62]. In a saturating mutagenesis experiment using a pan-KRAS inhibitor, mutations at the drug-binding site conferred resistance as expected [62]. KRAS mutations that impaired GEF activity, leading to an increase in targetable GDP-loaded KRAS, sensitized cells to inhibition with the pan-KRAS inhibitor. In patient tumors with KRAS G12C that were analyzed post-sotorasib treatment, 14% of tumors displayed an additional or alternative point mutation in KRAS [97]. Interestingly, some of the treatment-emergent KRAS secondary mutations that are not commonly observed in primary tumors, such as KRAS V8L and KRAS V14I, arise in patients who are relatively responsive to sotorasib (as defined by RECIST scores ≤ 30) [97]. Many of the novel mutations were observed at low subclonal frequencies, contributing to tumor heterogeneity [97,103]. This finding suggests that even low-frequency mutations can drive resistance to single-agent G12C inhibition. Time courses of G12C-mutant cell lines treated with increasing doses of G12C inhibitors demonstrated that the population frequency of rare resistance mutations increased over time [97]. In CRC patients co-treated with KRASi and EGFRi, low-frequency non-G12C KRAS mutations were detected in circulating tumor DNA, but were not well maintained over time [101]. Saturating mutagenesis screens and looking for point mutations in KRAS that produced resistance to CYPA recruiting compound RMC-6291 identified E31A, D33A, or E37A as resistance mutations that in combination decreased drug efficacy. These distinct sites reflected the interface between KRAS and CYPA, underscoring that the mechanism of drug action heavily impacts the probable mechanisms of escape selected for by cancer cells.

### 4.3. Copy Number Alterations in KRAS

A common mechanism of resistance to KRAS inhibitors is the genomic amplification of mutant KRAS, as this can alter the stoichiometric relationship between inhibitor and target. In a study of sotorasib-treated patients, 7% of patients in the study developed KRAS G12C amplifications [97]. KRAS G12C amplification was a consistent resistance mechanism that increased in frequency over the duration of the treatment of CRC patients and cell lines treated with combinations of KRAS and EGFR inhibitors [101]. Extrachromosomal double minutes are a mechanism by which genomic DNA can be circularized outside of chromosomes, allowing for uneven copy changes during cell division. MYCN and EGFR can be amplified in double minutes in neuroblastoma and breast carcinomas, respectively, raising the possibility that KRAS G12C amplifications may arise in a similar way in NSCLC and CRC [104,105]. In other post-treatment tumors, G12C mutations were undetectable, indicating the complete deletion of the drug target and activation of alternative oncogenic drivers as a mechanism of therapeutic escape.

Although the presence of very high doses of homozygous mutant KRAS can lead to senescence, a subset of heterozygous mutant KRAS-driven tumors select for the loss of the wildtype allele, suggesting that the wildtype allele may have a suppressive effect on mutant oncogene signaling [106,107]. In mutant KRAS cells, the presence of the wildtype allele can drive resistance to downstream pathway inhibitors including MEK [108].

### 4.4. Resistance Caused by Other Genes

Genetic changes upstream or downstream of KRAS represent another form of mutational resistance to targeted KRAS inhibitors. These mutations can often emerge as the activation of upstream signaling proteins and/or bypass mutations activating downstream effectors. Genetic studies seeking factors that can rescue the simultaneous deletion of KRAS, NRAS, and HRAS identified effector proteins including mutant RAFs, MEK, ERK, and KSR, and the deletion of NF1 as being capable of rescuing growth [109,110]. Additionally, the enforced expression of KSR decreased the efficacy of sotorasib in sensitive cells [110].

In KRAS inhibitor-resistant tumors, genetic alterations observed in upstream regulators included transmembrane growth factor receptors EGFR and FGFR2/3 as well as the KRAS-regulating GAP protein NF1 [102]. The intrinsic hydrolysis rate of G12C is inherently high; hence, the fact that the loss of the GAP NF1 can act as a resistance mechanism for KRAS inhibition suggests that NF1 may have KRAS-independent functions [4,56,111]. The downstream effectors of KRAS signaling that were mutated post-KRAS inhibition included activating lesions in PIK3CA, MEK1, BRAF, and RAF1 [51,68,97,112]. These mutations represent mechanisms of KRAS pathway activation independent of the KRAS gene itself.

Genome-wide CRISPR screens in untreated KRAS-mutant, NRAS-mutant, and wildtype cell lines identified several classes of synthetic lethalities with mutant KRAS. These included two processing enzymes involved in membrane targeting, RCE1 and ICMT, as well as RAS pathway members including SHOC2, RAF1, ERK, RSK1, and PREX1 [112]. CRISPR screens specifically investigating sotorasib resistance identified and validated ERK1/2, SHOC2, NRAS, CRAF, and BRAF as vulnerable targets that were synthetically lethal with KRAS inhibition [97]. AURKA is a KRAS G12C–CRAF interaction mediator that can bind directly to KRAS G12C and has also been implicated in resistance to KRASi in CRISPR screens [68]. Co-inhibition with AURKA inhibitor alisertib and G12C inhibitor ARS-1620 was highly synergistic in mouse models due to the defective activation of RAF by KRAS G12C [68]. The CRISPRi-mediated suppression of HRAS, NRAS, TSC1/2, and NF1 led to resistance to KRASi [113]. These studies highlight potential combinatorial targets for inhibition alongside KRASi.

Finally, treatment-emergent mutations were observed in genes from other signaling pathways. These included ALK, APC, CDKN2A/B, CTNNB1, IDH1/2, KEAP1, MET, MYC, NRAS, PTCH1, PTEN, RB1, RET, RICTOR, SMARCA4, and TP53 [50,97,102]. CRISPR screens using the KRAS G12C inhibitor adagrasib found the deletion of tumor suppressors KEAP1 and RB1 as mechanisms of resistance to KRAS inhibition in both in vitro and in vivo models [50]. Multiple CDKs and cyclins proved to be synthetically lethal with KRAS inhibition both in vitro and in vivo. sgRNAs against CAB39, DUSP4, and NRBP1 were enriched in sotorasib-resistant cells, suggesting that their loss confers a selective advantage during treatment [97]. CRISPRi screens using the precursor G12C inhibitor ARS-1620 in lung and pancreatic cancer cells identified a broad range of genes both core (SHP2) and ancillary (CDK4) to RAS function as collateral dependency genes whose targeting may potentiate KRASi in resistant cells [113]. These collectively point to another class of mutations that compensate for the inhibition of KRAS activity by upregulating alternative oncogenic pathways to KRAS. Finally, in vitro and in vivo CRISPR screens looking for genes causing resistance to the KRAS G12D inhibitor MRTX1133 also identified NF1, PTEN, KEAP1, and RB1 as genes whose loss decreased inhibitor efficacy. Conversely, the loss of EGFR, PTPN11, mTOR, and PIK3CA was selected against in the presence of a KRAS G12D inhibitor. Collectively, these activations and deletions present notable challenges to monotherapy treatment strategies because they can bypass the need for KRAS entirely. As such, a broader or combinatorial approach may prove necessary to combat resistances of these types.

## 5. Non-Mutational Resistance

### 5.1. Rapid Adaptive Resistance

While changes in the genome can impact the efficacy of cancer treatment strategies, resistance mechanisms can also arise without any genetic alterations. Short-term targeted inhibitor treatment can lead to a small subset of drug-insensitive cells in both patient tumors and laboratory models within 1–14 days, a timeframe less likely to induce permanent genetic alterations [114,115,116]. Drug-Tolerant Persister (DTP) cells can derive from pre-treatment stochastic expression variability in cell populations, or from treatment-induced changes in transcriptional profiles or epigenetic markers [115,117,118]. While resistant states can persist after drug holidays, they can also be reversed in the subpopulations of DTPs, with large accompanying transcriptional changes. The ability of persister cells to transition back and forth between sensitive and insensitive states demonstrates that the mechanisms underlying their drug resistance are not driven by permanent genetic changes [115,118,119]. In the early stages of treatment, non-mutational DTP cell lines can enter a quiescent non-growth state [115,118,120]. Quiescence can lead to insensitivity to drugs targeting essential growth pathways, as these pathways become less critical in dormant cells [115].

Cancer cells use non-mutational adaptations to resist a wide variety of single drugs or drug combinations, including inhibitors targeting BRAF, MEK, EGFR, and KRAS [118,119]. The mechanisms underlying non-mutational resistance to non-KRAS inhibitors include cellular stress response, immune evasion, anti-apoptosis, epithelial-to-mesenchymal transition (EMT), and altered metabolic pathways [115,118,121]. Non-mutational resistance to KRAS inhibition can be driven by a variety of factors, such as the upstream activation of the pathway by the alleviation of feedback, the reactivation of the pathway by the activation of a downstream member, the activation of alternative growth pathways, EMT, alterations in the tumor microenvironment, drug efflux pump activity, and others. Proposals for combination therapies aim to address many of these resistance mechanisms.

### 5.2. Reactivation of the RAS Pathway or Alternative Signaling

The inhibition of mutant KRAS rapidly decreases the phosphorylation of MEK and ERK, leading to a decrease in the expression of nuclear ERK-regulated genes including DUSP proteins, RSK, MYC, SPRY1, and SPRED1 (Figure 2) [50,62]. Active p-ERK drives negative feedback through its effector SPRY1, which inhibits GRB2 from activating SOS1, resulting in a subsequent decrease in KRAS signaling [96,122]. When KRAS is chemically inhibited, the ERK-mediated negative feedback is relieved, leading to the paradoxical reactivation of both the RAS signaling pathway and other parallel pathways governed by EGFR and SPRY. Sotorasib remains covalently bound to mutant KRAS, yet newly synthesized copies of mutant KRAS can still be activated by this feedback, along with its family members NRAS and HRAS, creating potential mechanisms of resistance to KRAS inhibitors [68,123].

KRAS inhibition can lead to the paradoxical reactivation of EGFR, driving resistance through KRAS-independent mechanisms. EGFR is itself frequently mutated or amplified in cancer patients, particularly in NSCLC [122]. Co-inhibiting KRAS and EGFR can shut down feedback loops activated by KRAS monotherapy [96,101,124]. The combinatorial inhibition of KRAS and EGFR in patients with KRAS G12C advanced colorectal cancer, a disease where KRASi monotherapy was not approved, shows promise in ongoing clinical trials.

KRAS inhibition leads to short-term decreases in the expression of genes related to glycolysis, mitotic spindle formation, G2M checkpoint, and the downstream targets of E2F, MYC, and mTOR signaling [50]. Additionally, the upregulation of the YAP/TEAD pathway has been implicated as a mechanism of resistance to KRAS inhibition [125,126]. Combination therapies with TEAD inhibitors synergize with KRAS inhibitors by blocking the cell cycle [127]. Combining the mTOR inhibitor vistusertib with MRTX849 treatment showed strong synergy against a panel of sensitive cell lines and xenograft models [50]. Of note, cells that become resistant to the combination of KRASi and EGFRi are sensitive to mTOR inhibition [101]. Finally, phosphoproteomic studies on NSCLC cells treated with KRAS inhibitors identified several additional activated pathways including the upregulation of ERBB2/3 and AXL [128]. Taken as a whole, KRAS-driven cancers have a plethora of alternative pathways by which their oncogenic programs in the absence of mutant KRAS can be restored.

In a recent study by Lv et al., the inhibition of a KRAS-inactivated protein quality control program resulted in wide-scale protein misfolding [82]. Sotorasib promoted the SEL1L-dependent ubiquitination of IRE1α, and cells that developed resistance to KRAS inhibition reactivated IRE1α via a MEK/ERK-independent, PI3K/AKT-dependent mechanism to functionally restore normal proteostasis. These findings suggest the IRE1α pathway may be a promising combinatorial target alongside direct KRAS inhibitors.

### 5.3. Epithelial-to-Mesenchymal Transition

The epithelial-to-mesenchymal transition (EMT) is a developmental specialization process commonly co-opted by cancer cells. EMT is particularly common in metastatic cancer cells since EMT increases cell motility, thereby enhancing the potential for intravasation and spread to other tissues [129]. A mesenchymal-like differentiation state can act as a resistance mechanism to KRAS inhibitors [126,128,130]. YAP, a pivotal EMT regulator gene, can restore oncogenicity in murine tumors that have lost KRAS [54,131]. Furthermore, the inhibition of KRAS can activate MRAS in a YAP-dependent manner, leading to a more mesenchymal marker profile [126].

### 5.4. Tumor Microenvironment

The tumor microenvironment plays a significant role in resistance to KRAS inhibitors. Oncogenic KRAS reprograms cellular metabolism towards aerobic glycolysis, resulting in increased lactate production, which is released into the extracellular space, acidifying the tumor microenvironment. This increased acidity can induce intracellular acidosis, hindering the antitumor functions of immune cells and thereby compromising immune surveillance, ultimately driving cancer progression. Monotherapy with the KRAS G12C inhibitor sotorasib increased the infiltration of T-cells, macrophages, and dendritic cells into tumors, sensitizing the TME to immunotherapy [48]. Notably, sotorasib has been shown to work synergistically with anti-PD-1 immune checkpoint inhibition in a syngeneic CT-26 colon cancer model. Combining the KRAS G12D inhibitor MRTX1133 with one or more immune checkpoint blockades was more effective than MRTX1133 as a single agent in a murine model of PDAC [132]. Unlike MRTX1133 monotherapy, which eventually allowed tumor escape leading to PDAC progression, the addition of either αCTLA-4 or αCTLA-4 with αPD-1 sustained tumor inhibition and facilitated immune-mediated cancer clearance [132]. Such findings suggest that combining KRAS inhibitors with immunotherapy could be a promising strategy to counteract resistance. Clinical trials testing the combination of KRAS inhibitors with checkpoint inhibitors in NSCLC and CRC are ongoing [101].

Gain-of-function screens of epigenetic regulators in KRAS-mutant PDAC models identified HDAC5 as a driver of resistance to KRAS inhibition within the tumor microenvironment [54]. Specifically, HDAC5 suppresses SOCS3, boosting CCL2 and CCL7 expression. This shifts the TME’s myeloid cells towards CCR2-expressing macrophages. In HDAC5-rich tumors, these macrophages increase TGFβ production, triggering pSMAD3/SMAD4 signaling in cancer cells, and bypassing the need for KRAS. This HDAC5-driven resistance mechanism uses paracrine actions to attract immune cells supporting cancer cell survival. The findings emphasize the TME’s role in resistance and suggest therapeutic strategies like combining TGFβ or CCL2/CCL7-CCR2 targeting with KRAS inhibitors [54].

Additionally, SHP2 inhibitors in combination with KRAS G12C inhibitors influenced the tumor microenvironment in murine models of PDAC. The combination therapy optimized immune responses by reducing immune-suppressing myeloid cells, increasing CD8+ T-cells, and making tumors more responsive to further treatments like PD-1 inhibitors. Further, SHP2 inhibition was found to directly reduce tumor blood supply. In light of these findings, combining SHP2 and KRAS G12C inhibitors offers a promising approach for treating KRAS-mutant tumors by reshaping the tumor’s immune landscape to counter resistance mechanisms [133].

### 5.5. Drug Efflux Pumps

One barrier to effective therapy is drug delivery. The efficacy of multiple KRAS G12Ci methodologies including the PROTAC LC-2 and covalent inhibitor ARS-1620 were impaired by high expression of Multi-Drug Resistance 1 (MDR1) activity, a classic drug efflux mechanism of resistance [134,135]. This transport protein exports small drug molecules out of target cells, preventing long-term inhibition. Cell lines resistant to PROTACs were rendered sensitive by genetic and pharmacological MDR1 inhibition, suggesting a combinatorial strategy involving KRAS PROTACs and MDR1 inhibitors [134].

### 5.6. Combination Therapies

A strategy for overcoming the drawbacks of resistance to KRASi monotherapy strategies is the application of synergistic drug combinations, particularly when they have non-overlapping mechanisms of resistance [136]. In immunocompetent murine models with KRAS G12C tumors, sotorasib induced antitumor immunity, supporting the rationale for ongoing combination trials combining KRAS inhibitors with immunotherapeutic agents [48]. Studies combining sotorasib with immunotherapeutics such as anti-PD-1 checkpoint blockade have shown promise [48,49,55]. Ongoing clinical trials of G12C inhibitors including sotorasib, adagrasib, LY3537982, GDC-6036, and JNJ-74699157 are investigating combination therapies with inhibitors of EGFR, MEK, PD-1, PD-L1, SHP-2, CDK4/6, mTOR, FGFR, and VEGF, as well as triple combinations of KRAS inhibitors with VEGFi plus chemotherapy, EGFRi plus chemotherapy, and EGFRi plus MEKi [4,137,138,139]. SHP2 inhibitors synergize with KRAS G12C inhibitors against sensitive mouse tumors, possibly because they prevent feedback reactivation driven by the loss of ERK-mediated EGFR repression when KRAS activity is abrogated and/or because SHP2 inhibitors drive KRAS to be in the G12C inhibitor-sensitive GDP-bound state of KRAS [50,133]. An alternative preclinical strategy being tested is combining inhibitors of TEAD proteins with KRASi, preventing feedback reactivation [125,127].

## 6. Conclusions

Clinically, the majority of KRAS-mutant cancers remain untreatable and those with treatable mutations are rarely cured. Therapeutic resistance to targeted inhibitors of KRAS remains a pressing concern to both clinicians and researchers. A range of promising strategies is being actively investigated and may prove to enhance long-term efficacy and decrease relapses. Investigating the molecular and genetic underpinnings of treatment resistance can provide a roadmap to a future where these cancers are broadly more treatable.

## Glossary

4EBP1	Eukaryotic Translation Initiation Factor 4E Binding Protein 1, encoded by the gene EIF4EBP1.
ALK	ALK Receptor Tyrosine Kinase, also known as Anaplastic Lymphoma Kinase.
Amplification	An increase in the number of copies in a region of the genome.
APC	APC Regulator Of WNT Signaling Pathway, also known as Adenomatous Polyposis Coli.
ARAF	A-Raf Proto-Oncogene, Serine/Threonine Kinase.
ARF	One of the proteins encoded by the gene Cyclin Dependent Kinase Inhibitor 2A, or CDKN2A. Also known as p19-ARF.
AURKA	Aurora Kinase A.
BiTE	Bispecific T-cell engager.
BRAF	B-Raf Proto-Oncogene, Serine/Threonine Kinase.
CAB39	Calcium-Binding Protein 39.
CAS9	CRISPR-associated protein 9, used together with CRISPR to cleave DNA.
CCL2	C-C Motif Chemokine Ligand 2.
CDK4	Cyclin-Dependent Kinase 4.
CDK6	Cyclin-Dependent Kinase 6.
CDKN2A	Cyclin-Dependent Kinase Inhibitor 2A, encodes p16-INK4A and ARF.
CDKN2B	Cyclin-Dependent Kinase Inhibitor 2B
COVID-19	Coronavirus disease of 2019, caused by the virus SARS-CoV-2
CRAF	Raf-1 Proto-Oncogene, Serine/Threonine Kinase, also known as RAF1.
CRC	Colorectal carcinoma or colorectal cancer.
CRISPR	Clustered Regularly Interspaced Short Palindromic Repeats, often used to knock out a gene of interest.
CRISPRi	Inhibitory form of CRISPR that uses dead Cas9 deficient at cutting DNA to recruit negative regulatory transcription factors to the promoter of a gene of interest.
CTLA-4	Cytotoxic T-Lymphocyte-Associated Protein 4, an immune checkpoint protein.
CTNNB1	Catenin Beta 1, also known as β-catenin.
CYCA	Cyclophilin A.
Cyclins	A family of proteins that bind to cyclin-dependent kinases and regulate cell cycle.
DC	Dendritic cell.
DTP	Drug tolerant persister cells.
DUSPs	Dual-Specificity Phosphatase protein family.
E2F	E2F Transcription Factor.
EGF	Epidermal Growth Factor.
EGFR	Epidermal Growth Factor Receptor.
EMT	Epithelial-to-mesenchymal transition.
ERK1	Mitogen-Activated Protein Kinase 3, also known as Extracellular Signal-Regulated Kinase 1, a protein encoded by the gene MAPK3.
ERK2	Mitogen-Activated Protein Kinase 2, also known as Extracellular Signal-Regulated Kinase 2, a protein encoded by the gene MAPK1.
FDA	United States Food and Drug Administration.
FGFR	Fibroblast Growth Factor Receptor 1.
G12Ci	An inhibitor that is selective for KRAS with the 12th amino acid altered from glycine to cysteine.
GAB1	GRB2-associated binding protein 1.
GAP	GTPase-activating protein, promotes hydrolysis of GTP to GDP
GDP	Guanosine diphosphate. KRAS is inactive when bound to GDP.
GEF	Guanine nucleotide exchange factor, promotes exchange of GDP to GTP
GM-CSF	Granulocyte-macrophage colony-stimulating factor, also known as colony-stimulating factor 2.
GRB2	Growth Factor Receptor Bound Protein 2.
GTP	Guanosine triphosphate. KRAS is active when bound to GTP.
HDAC5	Histone Deacetylase 5.
HLA	Human leukocyte antigens.
HRAS	HRas Proto-Oncogene, GTPase, also known as Harvey Rat Sarcoma Viral Oncoprotein.
ICMT	Isoprenylcysteine Carboxyl Methyltransferase.
IDH1	Isocitrate Dehydrogenase (NADP(+)) 1.
IDH2	Isocitrate Dehydrogenase (NADP(+)) 2.
IRE1α	Endoplasmic Reticulum To Nucleus Signaling 1, encoded by the gene ERN1.
KEAP1	Kelch-Like ECH-Associated Protein 1.
KRAS	KRAS Proto-Oncogene, GTPase, also known as Kirsten Rat Sarcoma Viral Proto-Oncogene and also abbreviated at K-Ras.
KRASi	An entity that inhibits KRAS.
KSR	Kinase Suppressor Of Ras 1, encoded by the gene KSR1.
MDR1	ATP-Binding Cassette Subfamily B Member 1, also known as Multidrug Resistance Protein 1, encoded by the gene ABCB1.
MEK	Mitogen-Activated Protein Kinase Kinase 1, encoded by the gene MAP2K1.
MET	MET Proto-Oncogene, Receptor Tyrosine Kinase.
MHC	Major histocompatibility complex.
MRAS	Muscle RAS Oncogene Homolog.
mTCR	Murine T-cell receptor.
mTOR	Mechanistic Target Of Rapamycin Kinase.
MYC	MYC Proto-Oncogene, BHLH Transcription Factor, also known as c-MYC and as Avian Myelocytomatosis Viral Oncogene Homolog.
NF1	Neurofibromatosis type 1.
NRAS	NRAS Proto-Oncogene, GTPase, also known as Neuroblastoma RAS Viral (V-Ras) Oncogene Homolog.
NSCLC	Non-small cell lung cancer.
p53	Tumor Protein P53, encoded by the gene TP53.
PD-1	Programmed Cell Death Protein 1.
PDAC	Pancreatic ductal adenocarcinoma.
PD-L1	CD274 Molecule, also known as Programmed Cell Death 1 Ligand 1, encoded by the gene CD274.
PTCH1	Patched 1.
SHP2	Protein Tyrosine Phosphatase Non-Receptor Type 11, encoded by the gene PTPN11.
PBL	Peripheral blood lymphocytes.
PIK3CA	Phosphatidylinositol-4,5-Bisphosphate 3-Kinase Catalytic Subunit Alpha, also known as PtdIns-3-Kinase Subunit P110-Alpha, encodes the p110α subunit of PI3K.
PIP2	Phosphatidylinositol 4,5-bisphosphate.
PIP3	Phosphatidylinositol-3,4,5-triphosphate.
PNA	Peptide nucleic acid, a synthetic molecule hybridizing DNA bases with a peptide polymer chain.
PREX1	Phosphatidylinositol-3,4,5-Trisphosphate Dependent Rac Exchange Factor 1.
PROTAC	Proteolysis targeting chimeras containing a domain binding the protein of interest linked to a domain that recruits an E3 ubiquitin ligase.
PTEN	Phosphatase And Tensin Homolog, also known as Phosphatidylinositol 3,4,5-Trisphosphate 3-Phosphatase And Dual-Specificity Protein Phosphatase PTEN.
RAB	RAB1A, Member of RAS Oncogene Family.
RAN	RAN, Member of RAS Oncogene Family.
RAS-GAP	RAS P21 Protein Activator 1, also known as p120GAP, encoded by the gene RASA1.
RB1	RB Transcriptional Corepressor 1, also known as Retinoblastoma 1.
RCE1	Ras Converting CAAX Endopeptidase 1.
RET	Ret Proto-Oncogene, also known as Rearranged During Transfection.
RGS	Regulator Of G Protein Signaling.
RHO	Rhodopsin.
RICTOR	RPTOR Independent Companion Of MTOR Complex 2.
RSK1	Ribosomal Protein S6 Kinase A1.
S6	Ribosomal Protein S6.
SARS-CoV-2	Severe acute respiratory syndrome coronavirus 2, the virus that causes COVID-19.
SHOC2	SHOC2 Leucine Rich Repeat Scaffold Protein.
siRNA	Small interfering ribonucleic acid, used to decrease expression of target messenger RNAs.
SMARCA4	SWI/SNF Related, Matrix Associated, Actin-Dependent Regulator Of Chromatin, Subfamily A, Member 4.
SOS1	SOS Ras/Rac Guanine Nucleotide Exchange Factor 1, also known as Son Of Sevenless Homolog 1.
SPRED1	Sprouty-Related EVH1 Domain Containing 1.
SPRY1	Sprouty RTK Signaling Antagonist 1.
Synthetic lethal/collateral dependency	Two genes whose alterations are lethal to cells only when they are in combination.
TCR	T-cell receptor.
TEAD	TEA Domain Transcription Factor 1.
TGFβ	Transforming Growth Factor Beta 1.
TME	Tumor microenvironment.
TSC1	TSC Complex Subunit 1, also known as Tuberous Sclerosis 1 Protein.
TSC2	TSC Complex Subunit 2, also known as Tuberous Sclerosis 2 Protein.
UGP2	UDP-Glucose Pyrophosphorylase 2.
VEGF	Vascular Endothelial Growth Factor.
YAP	Yes1-Associated Transcriptional Regulator.
